# A Low Albumin-to-Globulin Ratio Predicts a Poor Prognosis in Patients With Metastatic Non-small-cell Lung Cancer

**DOI:** 10.3389/fmed.2021.621592

**Published:** 2021-03-01

**Authors:** Ping Lu, Yifei Ma, Shaozhong Wei, Xinjun Liang

**Affiliations:** ^1^Department of Medical Oncology, Hubei Cancer Hospital, The Seventh Clinical School Affiliated of Tongji Medical College, Huazhong University of Science and Technology, Wuhan, China; ^2^Department of Gastrointestinal Oncology Surgery, Hubei Cancer Hospital, The Seventh Clinical School Affiliated With Tongji Medical College, Huazhong University of Science and Technology, Wuhan, China

**Keywords:** serum albumin, albumin to globulin ratio, prognosis, metastatic non-small-cell lung cancer, overall survival, progression-free survival

## Abstract

**Objective:** The serum albumin-to-globulin ratio (AGR) may be a useful prognostic factor for various cancers. This study aimed to evaluate the prognostic value of the AGR in patients with metastatic non-small-cell lung cancer (NSCLC).

**Methods:** A retrospective study was conducted on patients with stage IV NSCLC diagnosed in Hubei Cancer Hospital from July 2012 to December 2013. The formula for calculating the AGR was serum albumin/total protein-serum albumin. The chi-square test or Fisher's exact test was used to analyze the classified variables. The Kaplan-Meier method was used to analyze the overall survival (OS) rate, which was plotted with the R language. The impact of the AGR on OS and progression-free survival (PFS) was analyzed by a multivariate Cox proportional hazard model.

**Results:** A total of 308 patients were included in the study population. The optimal cutoff values for the AGR in terms of OS and PFS were 1.12 and 1.09, respectively, as determined by X-Tile software. Kaplan-Meier curve analysis showed that the difference in survival rate between patients with different AGR levels was statistically significant (*p* = 0.04). The OS of patients with a high AGR (≥1.12) was longer than that of patients with a low AGR (<1.12). PFS in the high AGR group were better than those in the low AGR group (16.90 vs. 32.07months, *p* = 0.008). The univariate and multivariate models proved that the AGR was an independent prognostic factor in metastatic NSCLC patients in terms of both OS (*p* = 0.009, hazard ratio [HR] = 0.55, 95% confidence interval [95% CI] = 0.35–0.86) and PFS (*p* = 0.004, HR = 0.55, 95% CI = 0.37–0.83).

**Conclusion:** The AGR, which is measured in routine clinical practice, is an independent prognostic factor in terms of OS and PFS in metastatic NSCLC and can serve as a prognostic tool for metastatic NSCLC.

## Introduction

As a result of early screening and treatment, as well as the aging of the population, the ratio of cancer survivors to cancer cases continues to increase (1). Over the past few decades, the overall incidence of cancer among women has been basically stable. It is estimated that there will be 1,762,450 new cancer cases and 606,880 cancer-related deaths in the United States in 2019. The incidence of lung cancer has continuously declined, and the incidence of lung cancer in men is declining twice as fast as that in women (2). Data from 2008 to 2014 indicate that among all cancers, pancreatic cancer (9%), esophageal cancer (19%), liver cancer (18%) and lung cancer (19%) have the lowest 5-year relative survival rates (2).

Although a variety of markers have been shown to predict the prognosis of cancer patients, the value of these markers in clinical practice is limited because they require invasive detection methods and/or are difficult to assess before treatment. The prognostic assessment of patients is crucial for the selection of better treatment strategies. In view of this, it is necessary to find prognostic indicators that are affordable, convenient and highly clinically feasible to predict and distinguish the prognosis of tumor patients according to their clinical characteristics (3).

Albumin and globulin have attracted wide attention as non-invasive prognostic factors of tumors. Albumin can be used to reflect the nutritional and systemic inflammatory status of cancer patients and can be used as a prognostic marker for diverse cancers, such as lung cancer (4), lymphoma (5), renal cell carcinoma (6), breast cancer (7, 8) and gastrointestinal cancers (9). Globulin, as one of the main cortisol-binding proteins, can participate in immune and inflammatory responses (10). Furthermore, the albumin-to-globulin ratio (AGR) has been widely recognized as a prognostic indicator of various cancers. Therefore, we retrospectively studied the clinical significance of the AGR in predicting the overall survival (OS) and progression-free survival (PFS) of patients with stage IV non-small-cell lung cancer (NSCLC).

## Methods

### Study Design

After approval by the Hubei Provincial Ethics Committee, this study retrospectively analyzed patients with stage IV lung cancer who were pathologically diagnosed in our hospital from June 2012 to December 2013. Fluorodeoxyglucose (FDG) PET/CT, contrast-enhanced MRI, contrast-enhanced CT and exfoliative cytology were used to identify the tumor stage according to the eighth edition of the TNM staging standard for non-small cell lung cancer. A total of 399 patients who met the inclusion requirements were initially identified. After applying the exclusion criteria, 91 patients were excluded. Patients with obvious infection within 2 weeks (*n* = 3); patients with other chronic infectious diseases, including tuberculosis (*n* = 25), chronic obstructive pulmonary disease (*n* = 23), chronic liver disease and/or severe liver insufficiency (*n* = 21), patients with chronic kidney disease and/or severe renal insufficiency (*n* = 11), autoimmune diseases (*n* = 2); and patients with other primary malignant tumors (*n* = 6) were excluded.

### Demographic and Clinical Variables

The clinicopathological data of patients, including age, sex, smoking, and drinking status, tumor location, family history of cancer, histology, local or distant metastases distant metastatic sites. Patients with one or more metastatic sites in the contralateral lobe, malignant tumor nodules in the pericardium or pleura, and malignant pericardial effusion or pleural effusion are considered stage M1a. M1b is used to describe patients with a single extrathoracic metastasis of a single organ. M1c is used to describe patients with multiple extrathoracic metastatic lesions. Therapeutic data were collected through medical records. Treatment options include chemotherapy, radiotherapy, anti-VEGF-therapy and EGFR-TKI therapy. The chemotherapy regimen mainly includes platin-vinorelbine, platin-gemcitabine, platin-pemetrexed and others. Other relevant laboratory indicators, such as alkaline phosphatase (ALP), lactate dehydrogenase (LDH), triglycerides (TG), AGR, prognostic nutritional index (PNI), lymphocyte-to-mononuclear cell ratio (LMR), neutrophil-to-lymphocyte ratio (NLR) and platelet-to-lymphocyte ratio (PLR), were measured at baseline before treatment and recorded in the patient information system. The calculation formula for the AGR was serum albumin (g/l)/(total protein–serum albumin), and that for PNI was 10 × serum albumin (g/dl) + 0.005 × lymphocyte count (per mm^3^).

### Follow-Up

The follow-up period was from the time of diagnosis in Hubei Cancer Hospital to December 2013 or last contact. The average follow up duration is 39.53 months (38.70–40.37). During this period, the patient underwent routine reexamination, such as blood tests and imaging examinations.

### Statistical Analysis

The optimal cutoff values of relevant laboratory indicators were determined through X-tile and converted into two categorical variables. The chi-square test and Fisher's exact test were used to analyze the relationship between categorical variables expressed as frequencies and percentages. The Kaplan-Meier method was used to analyze OS and PFS rates, and the R language was used to draw survival curves including 95% confidence intervals (95% CIs). Univariate and multivariate Cox regression models were used to analyze the hazard ratio (HR) and 95% CI. All tests were bilateral, and the differences were only considered statistically significant when *P* < 0.05. Statistical analysis was performed using SPSS Statistics 25.0 software.

## Results

### Patient Characteristics

The clinical and demographic characteristics of the patients pathologically diagnosed with stage IV NSCLC are summarized in Table 1. The majority of them (*n* = 216) were younger than 65 years old. Overall, 64.0% (*n* = 197) of the patients were male, while 36% (*n* = 111) were female. Of the 308 patients, 159 (51.6%) and 149 (48.4%) were never and current/ever smokers, respectively; 60.4% (*n* = 186) of patients were pathologically diagnosed with lung adenocarcinoma. 76 patients were M1a (24.7%), 65 patients were M1b (21.1%), 167 patients were M1c (54.2%). Treatment options include chemotherapy (*n* = 236, 76.6%), radiotherapy (*n* = 109, 35.4%), anti-VEGF-therapy (*n* = 18, 5.8%) and EGFR-TKI therapy (*n* = 86, 27.9%). The chemotherapy regimen mainly includes platin-vinorelbine (*n* = 100, 32.5%), platin-gemcitabine (*n* = 147, 47.7%), platin-pemetrexed (*n* = 111, 36.0%) and others (*n* = 68, 22.1%). Some of these patients received more than one chemotherapy regimen. 228 patients received chemotherapy as first-line treatment. The median number of first-line chemotherapy cycles was 4 [interquartile range (IQR): [2, 5] (not shown in the table)]. The median AGR was 0.87 (IQR: [0.77, 1.00]), and the median ALP was 75.6 (IQR: [63.7, 98.0]).

**Table 1:** Clinical parameters in 308 patients with metastatic NSCLC.

**Variable**	***N* (%)**	**Variable**	***N* (%) or Median [IQR]**
**Age**		**Chemotherapy regimen**	
<65	216 (70.1)	Platin-Vinorelbine	100 (32.5)
≥65	92 (29.9)	Platin-Gemcitabine	147 (47.7)
**Gender**		Platin-Pemetrexed	111 (36.0)
Male	197 (64.0)	Others	68 (22.1)
Female	111 (36.0)	**Chemotherapy**	
**Smoking Status**		No chemotherapy	72 (23.4)
Never	159 (51.6)	First-line treatment	228 (74.0)
Current or ever	149(48.4)	Second-line treatment	8 (2.6)
**Drinking Status**		**EGFR-TKI**	
Never	232 (75.3)	No	222 (72.1)
Current or ever	76 (24.7)	Yes	86 (27.9)
**Location**		**AGR**	0.87 (0.77–1.00)
Left	188 (61.0)		
Right	120 (39.0)		
**family history of cancer**		**ALP**	75.6 (63.7–98.0)
No	244 (79.2)		
Yes	64 (20.8)		
**Histology**		**LDH**	199.6 (167.9–256.7)
Adenocarcinoma	186 (60.4)		
Squamous cell carcinomas	74 (24.0)		
Others	48 (15.6)		
**Local or distant metastases**		**TG**	1.17 (0.91–1.62)
M1a	76 (24.7)		
M1b	65 (21.1)		
M1c	167 (54.2)		
**Distant metastatic site**		**PNI**	48.2 (43.2–52.5)
Lung contralateral	121 (39.3)		
Pleural	106 (34.4)		
Cerebral	82 (26.6)	**LMR**	2.55 (1.92–3.63)
Bones	125 (40.6)		
Adrenal	15 (4.9)		
Liver	32 (10.4)	**PLR**	156.1 (115.6–198.5)
Others	28 (9.1)		
**Radiotherapy**		**NLR**	3.12 (2.12–4.47)
No	199 (64.6)		
Yes	109 (35.4)		
**Anti-VEGF-therapy**			
No	290 (94.2)		
Yes	18 (5.8)		

### Cutoff Values for the Parameters

The median of the AGR is 0.87 (0.77–1.00). For OS analysis, X-tile determined the significant cutoff value of the AGR to be 1.12. Then, the patients were divided into two groups (AGR <1.12, AGR ≥ 1.12). Through this method, the optimal cutoff values for ALP, LDH, TG, PNI, LMR, NLR and PLR for OS analysis were determined, as shown in Table 2. We compared the AGR values based on clinical and demographic characteristics such as age, sex, smoking and drinking status, tumor location, family history of cancer, histology, local or distant metastases, distant metastatic sites and therapy regimens. The AGR was only significantly related to gender. The AGR was significantly related to certain laboratory indicators (such as the ALP, PNI, LMR, PLR, and NLR). For the PFS analysis, the cutoff value of the AGR was set to 1.09, and patients were divided into low AGR and high AGR groups. Similarly, the cutoff values of ALP (70.3), LDH (179.8), TG (0.87), PNI (53.2), LMR (1.92), NLR (2.1), and PLR (145.6) were identified for the PFS analysis.

**Table 2:** Statistical cutoff values for demographic and clinical variables.

**Variable**	**AGR**		**X2**	***P***
	**AGR <1.12**	**AGR≥1.12**		
	***N* = 258 (%)**	***N* = 50 (%)**		
**Age**			0.49	0.49
<65	183 (70.9)	33 (66.0)		
≥65	75 (29.1)	17 (34.0)		
**Gender**			3.75	0.05
Male	159 (61.6)	38 (76.0)		
Female	99 (38.4)	12 (24.0)		
**Smoking Status**			1.39	0.24
Never	137 (53.1)	22 (44)		
Current or ever	121 (46.9)	28 (56)		
**Drinking Status**			1.72	0.19
Never	198 (76.7)	34 (68)		
Current or ever	60 (23.3)	16 (32)		
**Location**			0.22	0.64
Left	156 (60.5)	32 (64.0)		
Right	102 (39.5)	18 (34.0)		
**Family history of cancer**			1.67	0.20
No	201 (77.9)	43 (86.0)		
Yes	57 (22.1)	7 (14.0)		
**Histology**			1.39	0.50
Adenocarcinoma	168 (65.1)	18 (36.0)		
Squamous cell carcinomas	54 (20.9)	20 (40.0)		
Others	36 (14.0)	12 (24.0)		
**Local or distant metastases**			0.91	0.64
M1a	65 (25.2)	11 (22.0)		
M1b	52 (20.2)	13 (26.0)		
M1c	141 (54.7)	26 (52.0)		
**Distant metastatic site**				
Lung contralateral	101 (39.1)	20 (40.0)	0.01	1.91
Pleural	91 (35.3)	15 (30.0)	0.51	0.47
Cerebral	69 (26.7)	13 (26.0)	0.01	1.91
Bones	108 (41.9)	17 (34.0)	0.07	0.30
Adrenal	13 (5.0)	2 (4.0)	0.10	0.76
Liver	29 (11.2)	3 (6.0)	1.24	0.27
Others	23 (8.9)	5 (10.0)	0.06	0.81
**Radiotherapy**			0.18	0.67
No	168 (65.1)	31 (62.0)		
Yes	90 (34.9)	19 (38.0)		
**Chemotherapy**			0.76	0.68
No chemotherapy	58 (22.5)	14 (28.0)		
First-line treatment	193 (74.8)	35 (70.0)		
Second-line treatment	7 (2.7)	1 (2.0)		
**Chemotherapy regimen**				
Platin-Vinorelbine	83 (32.2)	17 (34.0)	0.06	0.8
Platin-Gemcitabine	123 (47.7)	24 (48.0)	0.002	0.97
Platin-Pemetrexed	92 (35.7)	19 (38.0)	0.1	0.75
Others	59 (22.9)	9 (18.0)	0.58	0.45
**Anti-VEGF-therapy**			0.003	0.96
No	243 (94.2)	47 (94.0)		
Yes	15 (5.8)	3 (6.0)		
**EGFR-TKI**			2.92	0.08
No	181 (70.2)	41 (82.0)		
Yes	77 (29.8)	9 (18.0)		
**ALP**			4.12	0.04
<70.3	149 (41.6)	15 (36.6)		
≥70.3	209 (58.4)	26 (63.4)		
**LDH**			1.37	0.24
<269.6	281 (78.5)	28 (68.3)		
≥269.6	77 (21.5)	13 (31.7)		
**TG**			1.02	0.31
<1.05	117 (32.7)	17 (41.4)		
≥1.05	241 (67.3)	24 (58.6)		
**PNI**			13.31	<0.001
<51.2	275 (76.8)	41 (100)		
≥51.2	83 (23.2)	0 (0)		
**LMR**			6.04	0.01
<1.9	85 (23.7)	16 (39)		
≥1.9	273 (76.3)	25 (61)		
**PLR**			4.62	0.03
<122.5	110 (30.7)	5 (12.2)		
≥122.5	248 (69.3)	36 (87.8)		
**NLR**			7.32	0.007
<2.77	325 (90.8)	29 (70.7)		
≥2.77	33 (9.2)	12 (29.3)		

### Univariate Survival Analysis and Survival Curve Analysis

**Figure 1:**
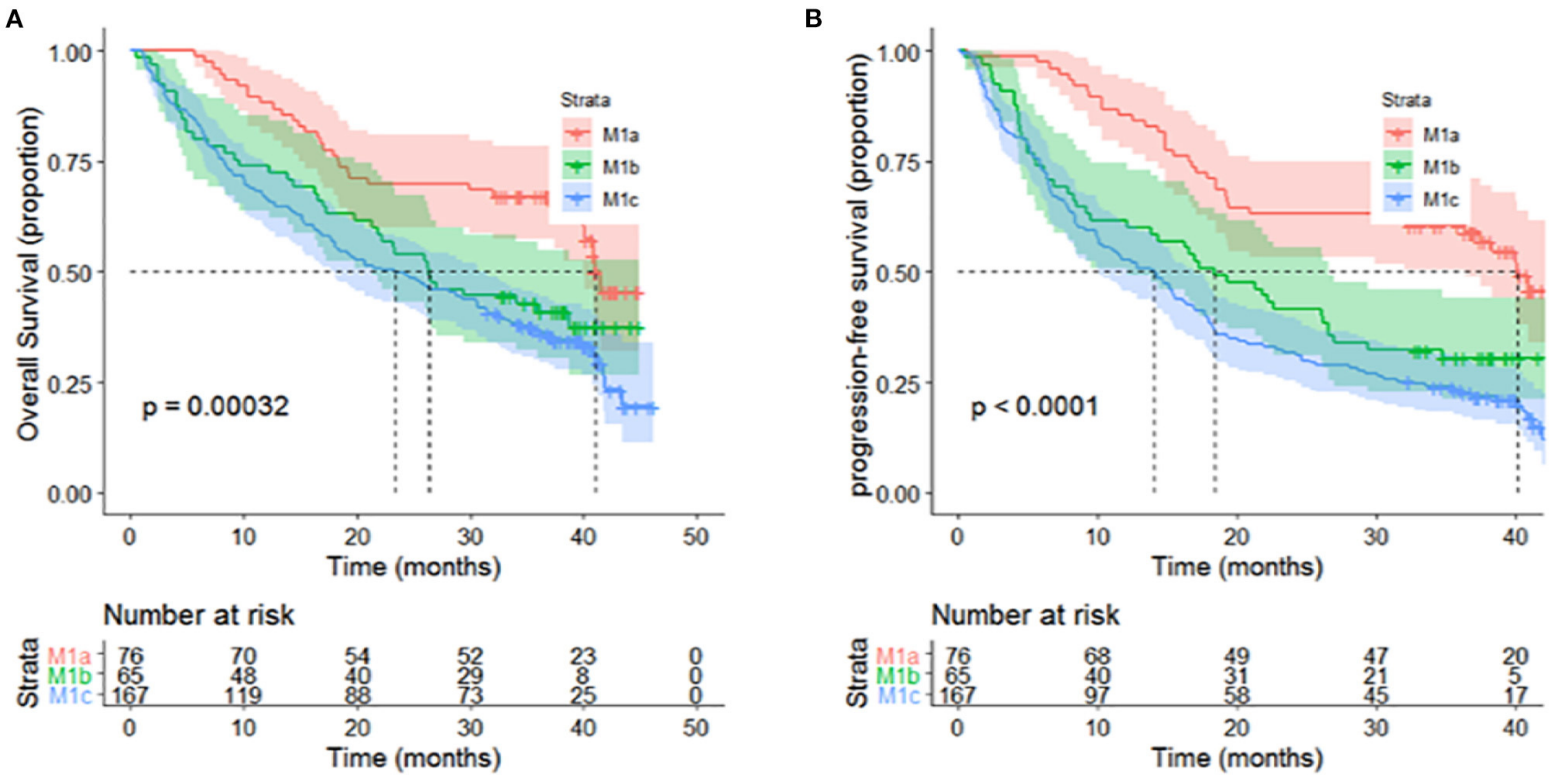
Different distant metastasis stages presented significant associations with **(A)** OS (*P* < 0.001) and **(B)** PFS (*P* < 0.0001) of patients with metastatic non-small-cell lung cancer in univariate analyses.

**Figure 2:**
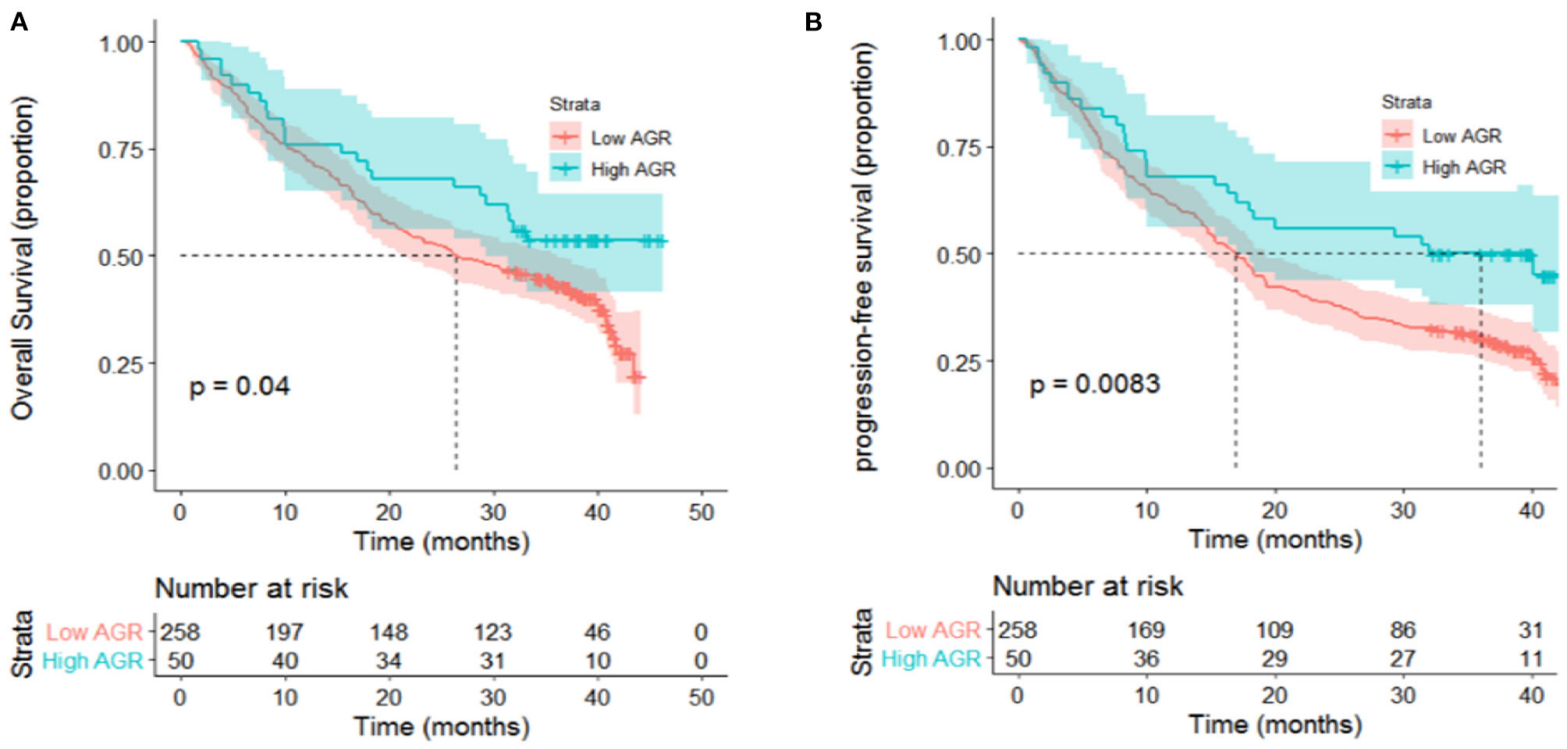
High levels of AGR presented significant associations with **(A)** OS (*P* = 0.04) and **(B)** PFS (*P* = 0.008) of patients with metastatic non-small-cell lung cancer in univariate analyses.

**Figure 3:**
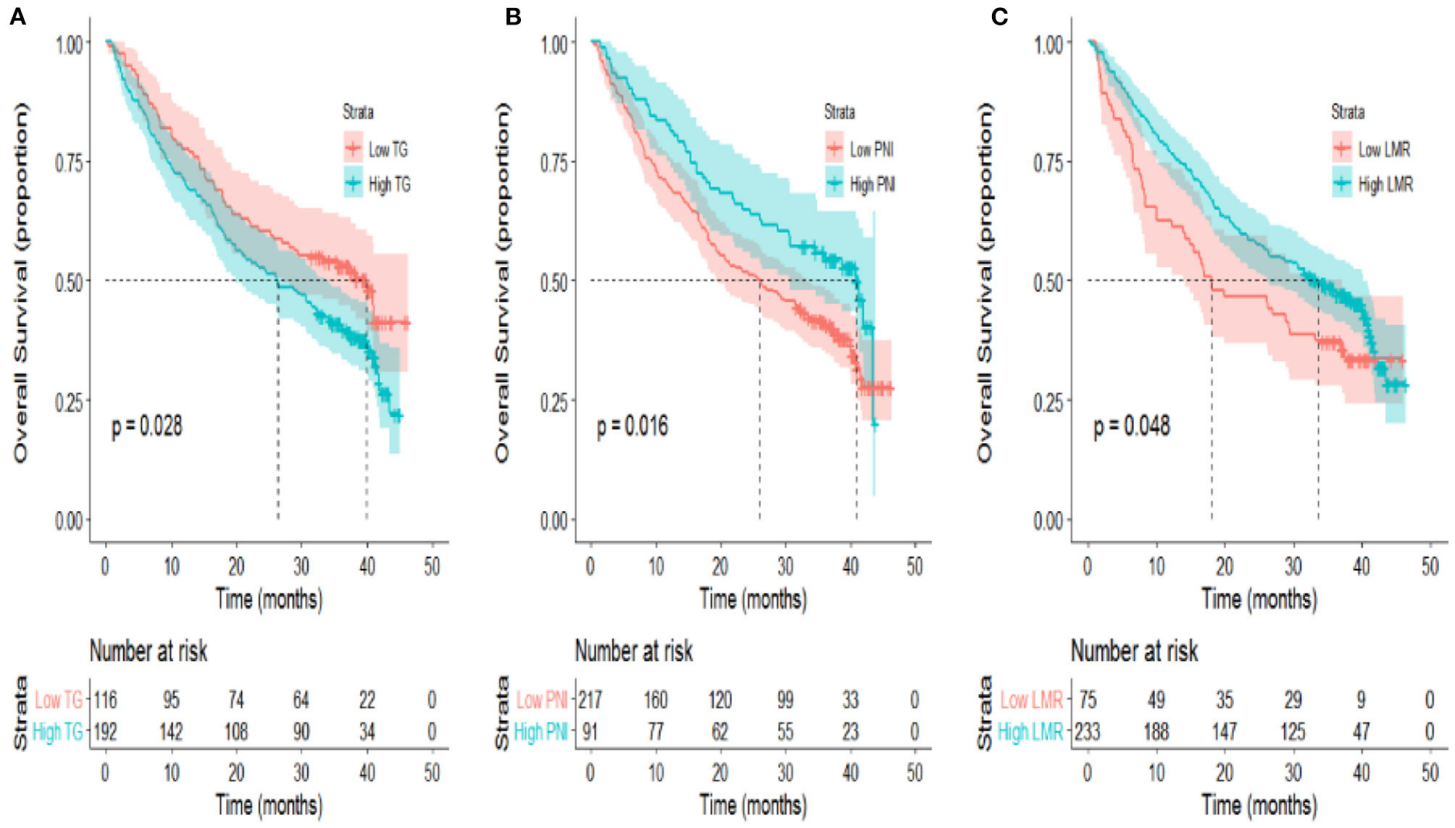
**(A)** TG (*P* = 0.028), **(B)** PNI (*P* = 0.016), **(C)** LMR (*P* = 0.048) presented significant associations with OS of patients with metastatic non-small-cell lung cancer in univariate analyses.

In the univariate analysis, age, sex, smoking and drinking status, tumor location, family history of cancer and histology were not associated with OS or PFS (Table 3). Local or distant metastases and some distant metastasis sites are related to prognosis. Compared with M1a category disease, patients with M1b category had poorer OS (HR = 1.83, *P* = 0.01) and PFS (HR = 2.05, *P* = 0.001) (Figure 1A). Compared with M1a category, M1c category disease had poorer OS (HR = 2.20, *P* < 0.001) and PFS (HR = 2.76, *P* < 0.001) (Figure 1B). Cerebral metastasis was associated with poor OS (HR = 1.76, P < 0.001) and PFS (HR = 1.91, *P* < 0.001) in univariate analysis. Bone metastasis was associated with poor OS (HR = 1.38, *P* = 0.03) and PFS (HR = 1.55, *P* = 0.001). Adrenal metastasis was only associated with poor PFS (HR = 1.69, *P* = 0.05). Patients who received chemotherapy as a first-line treatment seemed to have a longer OS (HR = 0.62, *P* = 0.04). However, specific chemotherapy regimens, epidermal-growth-factor receptor tyrosine-kinase inhibitors (EGFR-TKI) and anti-vascular endothelial growth factor (anti-VEGF) were not significantly associated with OS in patients with stage IV lung cancer (Table 3). EGFR-TKI was associated with PFS (HR = 1.37, *P* = 0.03) in univariate analysis. A low AGR was significantly associated with poor OS and PFS (Figure 2). The difference in survival rate between patients with different AGR levels was statistically significant (*p* = 0.04). The OS of patients with a high AGR (≥1.12) was longer than that of patients with a low AGR (<1.12). The PFS of patients with a high AGR (≥1.09) was longer than that of patients with a low AGR (<1.09) (16.9 vs. 32.07 months, respectively). In addition, LDH (*P* = 0.04) has also been shown to be associated with the prognosis of patients. TG (*P* = 0.03), PNI (*P* = 0.02) and LMR (*P* = 0.05) were predictive of OS but not PFS (Table 4, Figure 3).

**Table 3:** Univariate survival analysis of baseline characteristics for OS and PFS.

**Variable**	**OS**		**PFS**		**Variable**	**OS**		**PFS**	
	**HR**	***P***	**HR**	***P***		**HR**	***P***	**HR**	***P***
**Age**					Adrenal				
<65	1.00				No	1.00			
≥ 65	0.88	0.43	0.92	0.59	Yes	1.36	0.31	1.69	0.05
**Gender**					Liver				
Male	1.00				No	1.00			
Female	1.08	0.60	1.05	0.74	Yes	1.20	0.41	1.22	0.34
**Smoking Status**					Others				
Never	1.00				No	1.00			
Current or ever	0.84	0.25	0.97	0.80	Yes	0.83	0.49	0.70	1.17
**Drinking Status**					**Radiotherapy**				
Never	1.00				No	1.00			
Current or ever	0.97	0.86	1.19	0.26	Yes	1.36	0.04	1.48	0.005
**Location**					**Chemotherapy**				
Left	1.00				No chemotherapy	1.00			
Right	1.25	0.13	1.17	0.27	First-line treatment	0.62	0.04	0.82	0.22
**Family history of cancer**					Second-line treatment	1.28	0.54	1.78	0.13
No	1.00				**Anti-VEGF**				
Yes	0.93	0.69	1.00	0.98	No	1.00			
**Histology**					Yes	1.11	0.72	1.27	0.37
Adenocarcinoma	1.00				**EGFR-TKI**				
SCC	1.09	0.63	0.87	0.43	No	1.00			
Others	1.18	0.42	1.00	0.99	Yes	1.20	0.24	1.37	0.03
**Local or distant metastases**					**Chemotherapy regimen**				
M1a	1.00				Platin-Vinorelbine				
M1b	1.83	0.01	2.05	0.001	No	1.00			
M1c	2.20	<0.001	2.76	<0.001	Yes	0.74	0.07	0.92	0.58
**Metastatic site**					Platin-Gemcitabine				
Lung contralateral					No	1.00			
No	1.00				Yes	0.99	0.93	1.10	0.52
Yes	1.23	0.37	1.19	0.78	Platin-Pemetrexed				
Pleural					No	1.00			
No	1.00				Yes	0.95	0.76	1.06	0.69
Yes	1.31	0.07	1.21	0.17	Others				
Cerebral					No	1.00			
No	1.00				Yes	0.72	0.08	0.72	0.08
Yes	1.76	<0.001	1.91	<0.001					
Bones									
No	1.00								
Yes	1.38	0.03	1.55	0.001					

**Table 4:** Univariate survival analysis of inflammatory biomarkers for OS and PFS.

**Variable**	**OS**		**PFS**		**Variable**	**OS**		**PFS**	
	**HR**	***P***	**HR**	***P***		**HR**	***P***	**HR**	***P***
**AGR**					**PNI**				
Low	1.00				Low	1.00			
High	0.63	0.04	0.58	0.007	High	0.67	0.02	0.79	0.17
**ALP**					**LMR**				
Low	1.00				Low	1.00			
High	1.31	0.07	1.19	0.20	High	0.72	0.05	0.79	0.13
**LDH**					**PLR**				
Low	1.00				Low	1.00			
High	1.41	0.04	1.33	0.05	High	0.79	0.13	0.80	0.10
**TG**					**NLR**				
Low	1.00				Low	1.00			
High	1.41	0.03	0.81	0.18	High	1.28	0.11	1.29	0.12

### Multivariate Cox Regression Model

The influence of variables on OS and PFS was analyzed by a multivariate Cox proportional hazard model. Local or distant metastases, cerebral metastasis, bone metastasis, radiotherapy and chemotherapy, as well as laboratory indicators that were statistically significant in the univariate analysis model, were included in the multivariate Cox regression model (Table 5). Local or distant metastases (M1b, HR: 1.66; 95% CI: 1.00–2.75; *P* = 0.05. M1c, HR: 1.84; 95% CI: 1.10–3.08; *P* = 0.02), first-line chemotherapy treatment (HR: 0.60; 95% CI: 0.43–0.84; *P* = 0.003), AGR (HR: 0.55; 95% CI: 0.35–0.86; *P* = 0.009), TG (HR: 1.40; 95% CI: 1.02–1.94; *P* = 0.04) and PNI (HR: 0.56; 95% CI: 0.39–0.80; *P* = 0.02) were independent prognostic factors for OS. Furthermore, local or distant metastases (M1b, HR: 1.87; 95% CI: 1.18–2.97; *P* = 0.08. M1c, HR: 2.36; 95% CI: 1.50–3.71; *P* < 0.001), and the AGR was an independent prognostic factor for PFS (HR: 0.55; 95% CI: 0.37–0.83; *P* = 0.004).

**Table 5:** Multivariate analysis of baseline characteristics and inflammatory biomarkers for the prediction of OS and PFS.

**Variables**	**OS**		**Variables**	**PFS**	
	**HR (95% CI)**	***P***		**HR (95% CI)**	***P***
**Metastasis**			**Local or distant metastases**		
M1a			M1a		
M1b	1.66 (1.00–2.75)	0.05	M1b	1.87 (1.18–2.97)	0.08
M1c	1.84 (1.10–3.08)	0.02	M1c	2.36 (1.50–3.71)	<0.001
**Cerebral**			**Cerebral**		
No			No		
Yes	1.40 (0.98–2.00)	0.07	Yes	1.32 (0.95–1.82)	0.10
**Bones**			**Bones**		
No			No		
Yes	0.97 (0.67–1.39)	0.86	Yes	0.96 (0.70–1.32)	0.82
**Chemotherapy**			**Adrenal**		
No chemotherapy			No		
First-line treatment	0.60 (0.43–0.84)	0.003	Yes	1.39 (0.81–2.40)	0.23
Second-line treatment	1.22 (0.53–2.81)	0.65	**Radiotherapy**		
**Radiotherapy**			No		
No			Yes	1.17 (0.87–1.58)	0.29
Yes	1.22 (0.87–1.70)	0.25	**EGFR-TKI**		
**AGR**			No		
Low			Yes	1.09 (0.81–1.47)	0.57
High	0.55 (0.35–0.86)	0.009	**AGR**		
**LDH**			Low		
Low			High	0.55 (0.37–0.83)	0.004
High	1.12 (0.78–1.60)	0.55	**LDH**		
**TG**			Low		
Low			High	1.16 (0.87–1.55)	0.32
High	1.40 (1.02–1.94)	0.04			
**PNI**					
Low					
High	0.56 (0.39–0.80)	0.02			
**LMR**					
Low					
High	0.83 (0.59–1.18)	0.31			

## Discussion

Inflammation plays an important role in lung cancer and contributes to its occurrence and development (11–13). The AGR, which accounts for the values of both albumin and globulin, can be used as one of the inflammatory parameters to evaluate the systemic inflammatory status of the host (14). In addition to reflecting the nutritional status of the host, serum albumin can also be affected by inflammatory factors, which in turn reflects the level of inflammation in the body (15). Albumin production can be regulated by proinflammatory cytokines such as tumor necrosis factor (TNF) and interleukin-6 (IL-6). For example, TNF can inhibit the transcription of the albumin gene, leading to a low level of albumin in the host, which is conducive to tumor progression (16). Globulin levels may increase with the accumulation of acute phase proteins, including C-reactive protein and serum amyloid A. Some studies have found that the common variants of TNF receptor superfamily member 13B and other genes are strongly correlated with the increase in immunoglobulin, which suggests that globulin may be related to apoptosis and cancer progression (17). As mentioned above, both albumin and globulin could be involved in cancer progression in a variety of ways and play important roles. Based on this, it is reasonable to suggest that AGR, an index derived from albumin and globulin, can be used as one of the prognostic factors for cancer. A low AGR can reflect low albumin and/or high globulin levels in the host. In fact, the prognostic value of the AGR has been confirmed in many cancers, including NSCLC.

In this study, we investigated the prognostic value of the AGR, an index based on inflammation, in 308 patients with advanced metastatic NSCLC. In our study, we found that the AGR was not significantly associated with demographic characteristics but was significantly correlated with some laboratory indicators, such as the PNI, LMR, PLR and NLR. The patients were divided into two groups based on the best AGR cutoff value determined by X-Tile. In both the univariate and multivariate analysis models, the AGR was found to be significantly associated with the prognosis of patients with IV stage NSCLC. A low AGR predicted poor OS and PFS (HR: 0.55; 95% CI: 0.35–0.86; *P* = 0.009; HR: 0.55; 95% CI: 0.37–0.83; *P* = 0.004, respectively). Our results suggest that the AGR can be used as an index to predict the OS and PFS of patients with advanced metastatic NSCLC. In the future, more studies, especially prospective randomized studies, are needed to confirm the importance of the AGR in NSCLC.

For the specific clinical implementation of this inflammatory biomarker, the appropriate method for determining the optimal cutoff value will need to be considered. However, there is no consensus method for determining the cutoff value of the AGR. Different studies have used different methods to calculate the cutoff value of the AGR. Some studies used the median as the cutoff value; some studies used receiver operating characteristic (ROC) curve analysis to find the best cutoff value; and some studies even selected the appropriate cutoff value according to the quartiles. The cutoff value of the AGR ranges from 1.01 to 1.71 (18–20). In this study, using X-tile software, we determined the best cutoff value of the AGR to be 1.13. In the future, more verification cohorts are needed to determine whether these thresholds can be applied to other independent cohorts to further confirm the clinical prognostic value of the AGR.

## Conclusion

We report that a low AGR independently predicts poor OS and PFS in patients with IV NSCLC. The OS and PFS rates of patients with a low AGR are worse than those of patients with a high AGR. Therefore, the AGR is an important index for predicting the survival outcome of NSCLC and could assist in the selection of different treatment strategies.

## Data Availability Statement

The raw data supporting the conclusions of this article will be made available by the authors, without undue reservation.

## Ethics Statement

The studies involving human participants were reviewed and approved by Ethics Committee of Hubei Cancer Hospital. The patients/participants provided their written informed consent to participate in this study.

## Author Contributions

XL and SW: study design and final approval. PL and YM: performed study and literature search. PL: data collection, analysis of data, and drafting the work. All authors contributed to the article and approved the submitted version.

## Conflict of Interest

The authors declare that the research was conducted in the absence of any commercial or financial relationships that could be construed as a potential conflict of interest.
